# Penile Metastasis From Prostatic Adenocarcinoma: A Case Report

**DOI:** 10.7759/cureus.77039

**Published:** 2025-01-06

**Authors:** José Laert, Tomás França Santana, Nuno Lupi Manso

**Affiliations:** 1 Radiology Department, Hospital CUF Tejo, Lisbon, PRT

**Keywords:** magnetic resonance imaging, penile metastasis, penis cancer, prostatic adenocarcinoma, urologic oncology

## Abstract

Penile metastasis is a rare occurrence, particularly when it presents as the first clinical manifestation of an underlying malignancy. Most cases of penile metastasis arise from genitourinary cancers, particularly prostate and bladder carcinomas. This report discusses the case of a 68-year-old male patient who presented with hematuria and penile pain, accompanied by penile induration and tenderness on palpation during physical examination. Magnetic resonance imaging (MRI) revealed a locally aggressive prostatic tumor, confirmed as adenocarcinoma on biopsy, along with multiple nodular lesions in the corpus spongiosum and corpora cavernosa of the penis, exhibiting intermediate T2 signal intensity, heterogeneous enhancement on contrast-enhanced imaging, and significant diffusion restriction. These findings were strongly suggestive of penile metastasis in the context of disseminated prostate cancer, and the patient was managed with palliative hormone therapy and radiation to alleviate symptoms.

This article highlights the rare presentation of penile metastasis as the initial sign of advanced prostate adenocarcinoma, exploring the clinical challenges associated with its diagnosis and the underlying mechanisms of metastatic spread. Emphasis is placed on the critical role of imaging in detecting penile involvement, which can often be challenging to diagnose through clinical examination alone. Despite its rarity, penile metastasis as the first manifestation of cancer requires early recognition and timely intervention. This report underscores the importance of considering penile metastasis in patients, even in the absence of a prior history of malignancy, and emphasizes the need for a comprehensive approach that includes advanced imaging techniques and palliative care strategies to optimize patient outcomes, which are generally poor in this clinical context.

## Introduction

Penile metastasis is an exceptionally rare occurrence and often overlooked manifestation of disseminated disease. While metastatic involvement of the penis is uncommon, the presence of secondary tumors in this organ raises important clinical and diagnostic challenges. Despite the penis's rich vascularization and anatomical proximity to major pelvic organs, the occurrence of metastasis remains limited, likely due to unique anatomical and physiological barriers [1]. The exact mechanism of metastasis is not yet understood, although it has been proposed to occur through direct invasion or retrograde spread via venous, lymphatic, or arterial pathways [2]. Prostate adenocarcinoma, the most prevalent malignancy of the male genitourinary tract, is among the most common primary sources of penile metastases [3]. The lesions most commonly present with symptoms such as painless penile nodules, induration, and malignant priapism [4]. Penile metastasis is often identified in the clinical setting of disseminated disease, and it typically carries a very poor prognosis [3].

This report presents a rare case of a 68-year-old male patient diagnosed with penile metastasis as the first manifestation of advanced prostate adenocarcinoma, highlighting the complexities of its diagnosis. We aim to provide a comprehensive exploration of the pathophysiology, mechanisms of metastatic spread, clinical presentation, and diagnostic imaging features of penile metastasis. Furthermore, we will examine therapeutic and prognostic implications for patients with penile involvement, with an emphasis on the rarity of its occurrence as the initial manifestation of primary neoplasia.

## Case presentation

A 68-year-old male patient was brought to the emergency department with frank hematuria and penile pain. His medical history included benign prostatic hyperplasia (BPH) without follow-up. On physical examination, the patient exhibited induration of the penile shaft, with pain upon palpation of the corpora cavernosa and perineal region. Laboratory workup revealed anemia and a prostate-specific antigen (PSA) level of 17,6 ng/mL. 

**Figure 1:**
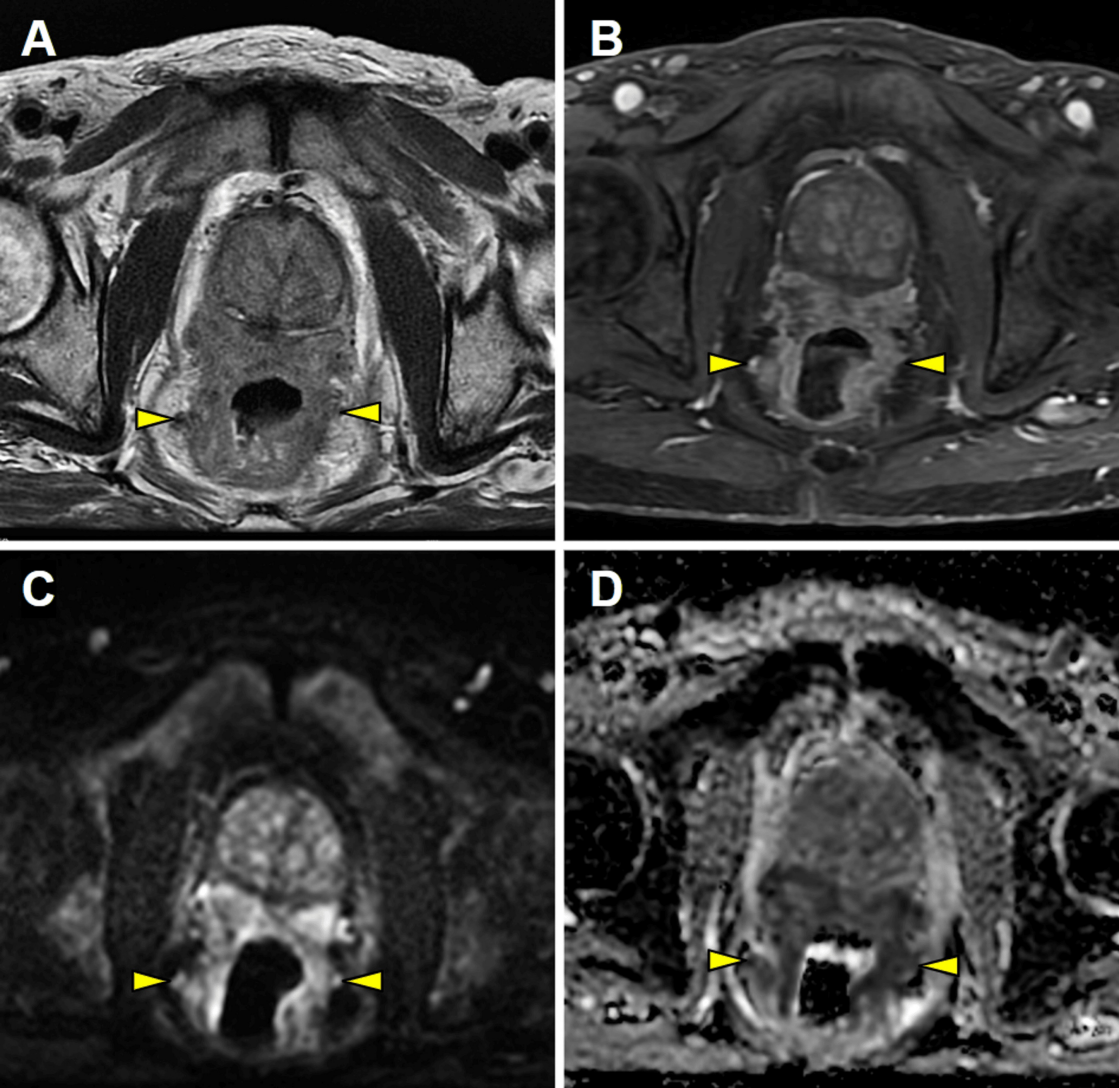
MRI study demonstrating a locally aggressive prostatic tumor MRI: magnetic resonance imaging Axial T2-weighted imaging (T2-WI) (A), gadolinium-enhanced fat-suppressed T1-weighted imaging (FS T1-WI) (B), diffusion-weighted imaging (DWI) (C), and apparent diffusion coefficient (ADC) map (D) reveal a large, locally aggressive prostatic tumor with invasion of the seminal vesicles, lymphovascular bundles, and rectal walls (arrowheads). The lesion demonstrates intermediate signal intensity on T2-WI, hypervascularity on gadolinium-enhanced FS T1-WI, and marked diffusion restriction, as evidenced by high signal intensity on DWI and corresponding low signal intensity on the ADC map

Magnetic resonance imaging (MRI) revealed a large and locally aggressive prostatic tumor arising from the peripheral glands with invasion of the seminal vesicles, lymphovascular bundles, and rectal walls. The lesion demonstrated intermediate signal intensity on T2-weighted images (T2-WI), hypervascularity on gadolinium-enhanced fat-saturated T1-weighted images (FS T1-WI), and marked diffusion restriction, as evidenced by high signal intensity on diffusion-weighted imaging (DWI) and corresponding low signal intensity on the apparent diffusion coefficient (ADC) map (Figure 1).

**Figure 2:**
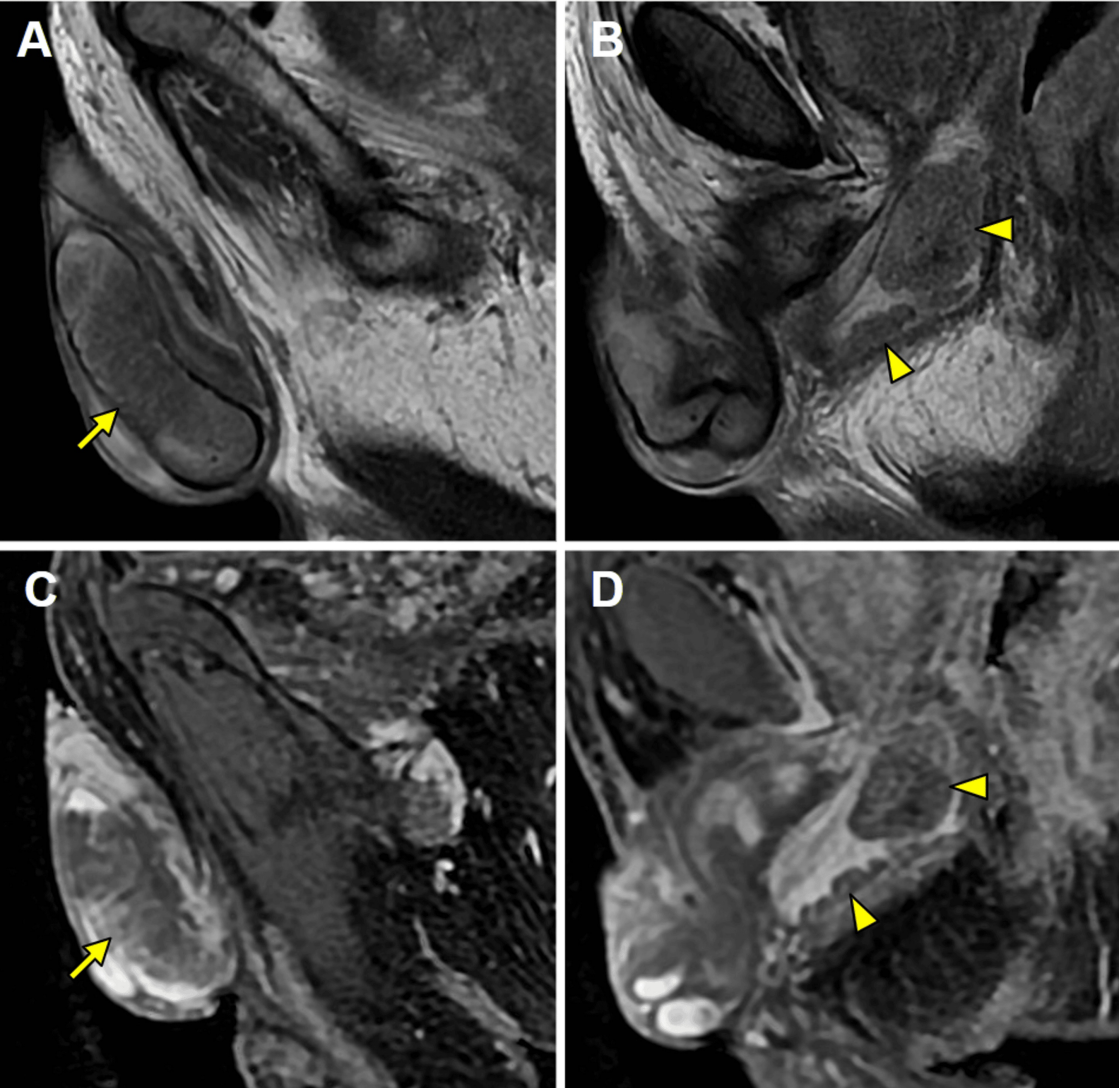
MRI study demonstrating multiple metastatic penile lesions. MRI: magnetic resonance imaging Sagittal T2-weighted imaging (T2-WI) (A-B) and gadolinium-enhanced fat-suppressed T1-weighted imaging (FS T1-WI) (C-D) reveal multiple lesions involving the corpora cavernosa (arrows; A, C) and the bulbospongiosum region of the penis (arrowheads; B, D). These lesions exhibit intermediate signal intensity on T2-WI and heterogeneous enhancement on gadolinium-enhanced FS T1-WI

**Figure 3:**
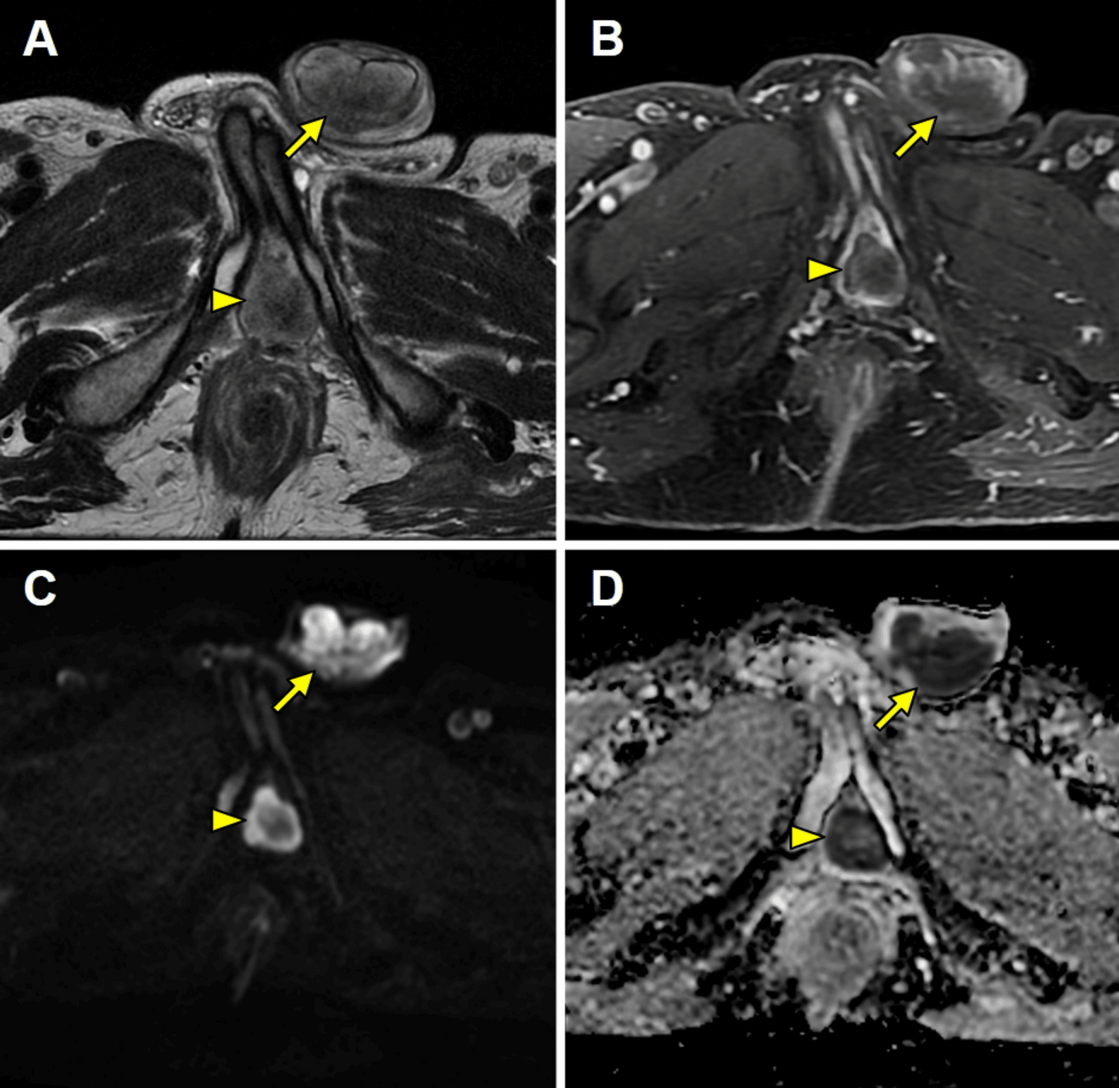
MRI study demonstrating multiple metastatic penile lesions MRI: magnetic resonance imaging Axial T2-weighted imaging (T2-WI) (A), gadolinium-enhanced fat-suppressed T1-weighted imaging (FS T1-WI) (B), diffusion-weighted imaging (DWI) (C), and apparent diffusion coefficient (ADC) map (D) reveal multiple lesions involving the corpora cavernosa and corpus spongiosum with disruption of the tunica albuginea (arrows) and the bulb of the penis (arrowheads). The lesions exhibit intermediate signal intensity on T2-WI, heterogeneous enhancement on gadolinium-enhanced FS T1-WI, and marked diffusion restriction, as indicated by high signal intensity on DWI and corresponding low signal intensity on the ADC map.

Additionally, the penis showed multiple scattered nodular lesions in the corpus spongiosum extending from the bulb, and in the corpora cavernosa, with disruption of the tunica albuginea. The lesions exhibited intermediate signal intensity on T2-WI and marked diffusion restriction, with high signal intensity on DWI and corresponding low signal intensity on the ADC map, and heterogeneous enhancement on contrast-enhanced imaging (Figures 2-3). In the setting of disseminated prostatic cancer, a presumptive diagnosis of penile metastasis was established.

**Figure 4:**
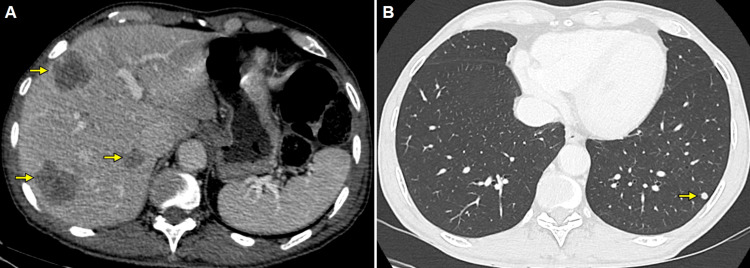
CT showing multiple metastatic lesions in the liver and lungs Iodine contrast-enhanced computed tomography (CT) reveals multiple metastatic lesions in the liver, characterized as hypovascular nodules with slight peripheral enhancement (arrows, A), and in the lung, presenting as scattered solid nodules (arrow, B)

A transrectal ultrasound-guided prostate biopsy confirmed prostate cancer, and his Gleason score was 10 (5+5). Further disease staging with iodine contrast-enhanced computed tomography (CT) revealed metastatic deposits in the liver and lungs (Figure 4).

The patient was selected for hormone therapy and received palliative radiation therapy to alleviate the pain caused by the penile metastasis, which was effective in reducing the pain. However, he later developed ulceration of the penile skin, which was managed conservatively. Despite these interventions, the patient passed away seven months after the diagnosis due to liver failure, likely secondary to the spread of the disease.

## Discussion

Metastatic disease involving the penis is a rare occurrence, despite its extensive vascular network. The seed-and-soil hypothesis suggests that metastasis depends not only on the traits of cancerous cells but also on the receptiveness of the host tissue. The penis's robust arterial and venous connections likely hinder tumor cell implantation, making it a less favorable "soil." However, conditions that disrupt venous or lymphatic drainage, such as tumors in nearby genitourinary structures or widespread pelvic disease, may create a more favorable environment for metastatic seeding [1].

The majority originate from the genitourinary tract, particularly prostatic adenocarcinoma and bladder carcinoma, while gastrointestinal tumors are less common [2,3]. Several other primary sites have also been reported, including the kidneys, lungs, testes, and hematologic system [1].

Several mechanisms of penile metastasis have been proposed, including retrograde venous, lymphatic, arterial, direct extension, and iatrogenic spread. The most common route is retrograde venous spread, due to the communication between pelvic venous plexuses and the penile dorsal venous system. This explains why most penile metastases originate from the prostate, bladder, and rectosigmoid area, with a preference for the corpora cavernosa. Retrograde lymphatic spread is another route, as the penis shares lymphatic drainage with the bladder, prostate, and lower rectum. Direct tumor extension from nearby organs may also occur, particularly in large, advanced tumors, though this is less common since most secondary tumors are found in the distal penile shaft. Arterial spread is rare, and iatrogenic spread due to urinary tract instrumentation is unlikely to be a significant cause [2].

Penile metastases present with a wide range of clinical signs, often characterized by painless, palpable nodules and induration, or malignant priapism, a persistent erection resulting from the presence of a tumor [4]. Penile metastasis generally develops within deeper tissues and is uncommon as a superficial skin presentation [1]. Some patients may also experience hematuria and penile pain [5].

Penile metastasis should be distinguished from malignant primary lesions like squamous cell carcinoma, and from infectious diseases such as tuberculosis, and syphilitic lesions, as well as other benign conditions, particularly Peyronie's disease [1]. In contrast to penile metastases, which typically manifest as multiple discrete masses in the corpora cavernosa and corpus spongiosum, primary penile cancers are most often solitary, ill-defined, infiltrating tumors [5]. The most common penile locations for metastasis are the glans and corpora cavernosa [6], although our case features diffuse involvement, including the bulb.

Imaging findings of penile metastasis can often be nonspecific. However, MRI is a valuable tool for diagnosing and staging the condition, enabling the identification of involvement of the corpora cavernosa and corpus spongiosum, spread through the tunica albuginea, and infiltration into the urethra and surrounding tissues, features that may be difficult to detect during a physical exam [4]. These lesions typically appear iso- or hypointense on T1-WI, often indistinguishable from adjacent tissues. Conversely, on T2-WI, they display a heterogeneous pattern with low to intermediate signal intensity, contrasting distinctly with the high signal intensity of the corpora cavernosa and corpus spongiosum [1]. The lesions are typically described as heterogeneous on contrast-enhanced imaging and show significant diffusion restriction on diffusion-weighted imaging. Metastatic disease may show involvement of the corpora without affecting the skin, which is unusual for squamous cell carcinomas, the most common primary penile malignancy, as they typically arise in the skin and secondarily invade the corpora. When multifocal corporal disease is identified in the context of a known malignancy, metastatic disease should be strongly considered [4].

When these lesions occur synchronously, they are often linked to disseminated malignancy and typically have a poor prognosis, with survival for patients with metastatic penile cancer often lasting no longer than one year [3,7]. In some cases, metastatic involvement of the penis may occur metachronously, sometimes years after the primary tumor has been diagnosed and treated [1]. As demonstrated in our case, penile involvement as the initial presentation of primary cancer is exceptionally rare.

The treatment of penile metastases typically follows the protocols of palliative care, as these patients often have widespread disease and a significantly reduced life expectancy. The approach may involve radiation therapy for pain relief, chemotherapy, or hormonal therapy, depending on the tumor's sensitivity to castration. Pain management, preservation of quality of life, and control of local complications are the primary goals of treatment [8].

## Conclusions

This case highlights the importance of a thorough diagnostic approach to penile metastasis, reminding clinicians to include this rare condition in the differential diagnosis for patients with suspected genitourinary malignancies or atypical penile presentations. Despite its rarity, evaluating the external genital area in such patients is crucial, as it is often overlooked. The mechanisms of metastatic spread to the penis reflect the intricate interplay of anatomical and physiological factors that contribute to its rarity.

Imaging, particularly MRI with its potential for evaluating soft tissues, is essential for identifying penile involvement and distinguishing it from primary penile cancer or other benign lesions. Given the poor prognosis associated with penile metastasis, particularly when it is the first clinical indication of disseminated disease, as in the reported case, timely recognition and intervention are crucial for improving the patient’s quality of life.
